# A Review of Pharmacology, Toxicity and Pharmacokinetics of 2,3,5,4′-Tetrahydroxystilbene-2-O-β-D-Glucoside

**DOI:** 10.3389/fphar.2021.791214

**Published:** 2022-01-05

**Authors:** Cheng Wang, Shu Dai, Lihong Gong, Ke Fu, Cheng Ma, Yanfang Liu, Honglin Zhou, Yunxia Li

**Affiliations:** State Key Laboratory of Southwestern Chinese Medicine Resources, Key Laboratory of Standardization for Chinese Herbal Medicine, Ministry of Education, School of Pharmacy, Chengdu University of Traditional Chinese Medicine, Chengdu, China

**Keywords:** 2,3,5,4′-tetrahydroxystilbene-2-O-β-D-glucoside, anti-inflammation, antioxidant, neuroprotection, cardiovascular protection, pharmacological and pharmacokinetic properties, toxicity

## Abstract

*Polygonum multiflorum* Thunb. (*He-shou-wu* in Chinese), a Chinese botanical drug with a long history, is widely used to treat a variety of chronic diseases in clinic, and has been given the reputation of “rejuvenating and prolonging life” in many places. 2,3,4′,5-tetrahydroxystilbene-2-O-β-D-glucoside (TSG, C_20_H_22_O_9_) is the main and unique active ingredient isolated from *Polygonum multiflorum* Thunb., which has extensive pharmacological activities. Modern pharmacological studies have confirmed that TSG exhibits significant activities in treating various diseases, including inflammatory diseases, neurodegenerative diseases, cardiovascular diseases, hepatic steatosis, osteoporosis, depression and diabetic nephropathy. Therefore, this review comprehensively summarizes the pharmacological and pharmacokinetic properties of TSG up to 2021 by searching the databases of Web of Science, PubMed, ScienceDirect and CNKI. According to the data, TSG shows remarkable anti-inflammation, antioxidation, neuroprotection, cardiovascular protection, hepatoprotection, anti-osteoporosis, enhancement of memory and anti-aging activities through regulating multiple molecular mechanisms, such as NF-κB, AMPK, PI3K-AKT, JNK, ROS-NO, Bcl-2/Bax/Caspase-3, ERK1/2, TGF-β/Smad, Nrf2, eNOS/NO and SIRT1. In addition, the toxicity and pharmacokinetics of TSG are also discussed in this review, which provided direction and basis for the further development and clinical application of TSG.

## 1 Introduction

*Polygonum multiflorum* Thunb. (PM), a famous traditional Chinese botanical drug, is often used as a tonic or anti-aging agent in some regions of Asia (Ling and Xu, 2016). For its clinical application, raw and prepared PM are differentiated, and both of them are collected in the *Chinese Pharmacopoeia* 2020. It has been reported that raw PM has the efficacies of detoxification, eliminating carbuncle, and relaxing bowel, while prepared PM can tonify the liver and kidney, boost essence blood, strengthen muscles and bones, and eliminate turbid blood and reduce lipids. In fact, PM is often utilized in compound preparations, such as Shouwu Pills, Yangxue Anshen Tablets, Yangxue Shengfa Capsules, and Shenbao Tablets. These preparations and health products containing PM are not only sold in the Chinese market, but also available far from foreign markets. Current research has showed that PM contains multiple components such as anthraquinones, stilbene glycosides, flavonoids, phospholipids, and phenolics (Liu et al., 2018). Among them, 2,3,5,4′-tetrahydroxystilbene-2-O-β-D-glucoside (TSG) is the most unique and important water-soluble ingredient of PM.

**Graphical Abstract:**
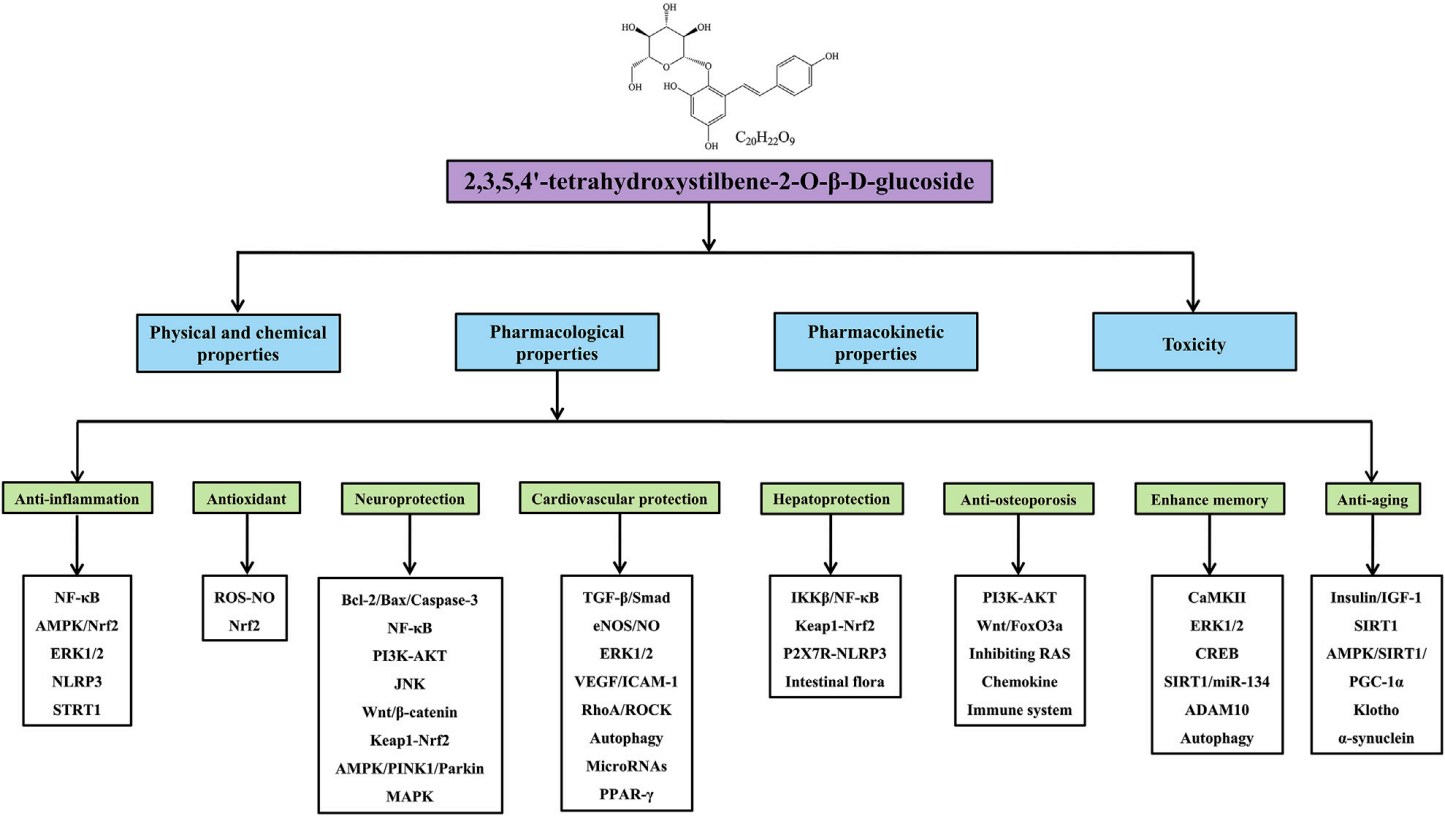


Modern pharmacological research has shown that TSG has significant pharmacological effects, including anti-inflammation (Chin et al., 2016; Li et al., 2018), antioxidation (Wen et al., 2020; Wu et al., 2020), neuroprotection (Gao et al., 2020; Tong et al., 2020), cardiovascular protection (Hu et al., 2019; Jia et al., 2019), hepatoprotection (Wang et al., 2020a; Zhang et al., 2020), anti-osteoporosis (Zheng et al., 2017; Zhang et al., 2018a), memory enhancement (Hou et al., 2011; Chen et al., 2016), and anti-aging (Zhang et al., 2013c; Fan et al., 2021), which largely contribute to the prevention and treatment of various diseases, such as inflammatory diseases, neurodegenerative diseases, cardiovascular diseases, liver injury, osteoporosis, and diabetic nephropathy. More importantly, nuclear factor kappa-B (NF-κB) (Lin et al., 2015b), Adenosine 5′-monophosphate (AMP)-activated protein kinase (AMPK) (Ning et al., 2018), Phosphatidylinositide 3-kinase (PI3K)-protein kinase B (AKT) (Zhang et al., 2013b), reactive oxygen species (ROS)-nitric oxide (NO) (Tao et al., 2011), B-cell lymphoma-2 (Bcl-2)/Bcl-2-associated X protein (Bax)/Caspase-3 (Zhou et al., 2020), and transforming growth factor-β (TGF-β)/Smad signaling pathways (Yao et al., 2015), have been shown to mediate the therapeutic effects of TSG. However, there is a lack of a comprehensive summary of TSG currently. Therefore, this article provides a systematic review about the physical/chemical properties, pharmacological effects, toxicity, and pharmacokinetic properties of TSG up to 2021 through searching the Web of Science, PubMed, ScienceDirect and CNKI databases, with the aim to provide guidance and evidence for the further development and clinical application of TSG.

## 2 Physical and Chemical Properties of TSG

**FIGURE 1:**
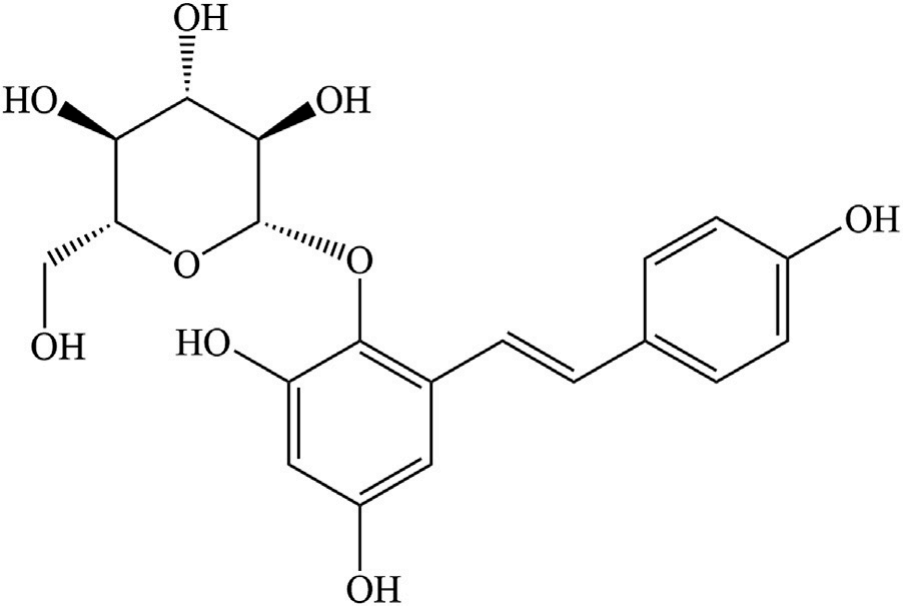
Chemical structure of TSG.

TSG (C_20_H_22_O_9_, Figure 1) is a polyhydroxy stilbene compound extracted from the traditional tonic TCM PM (Lin et al., 2015a). It was first discovered from the tuberous roots of PM by Japanese scientists in 1975 (Hata et al., 1975). Since then, there has been increased attention and research on TSG. More importantly, as the most predominant and unique active ingredient in PM, TSG is considered as a quality detection index in *Chinese Pharmacopoeia* 2020. It is specified that the TSG content in raw PM should not be less than 1.00% and that in prepared PM less than 0.70% (Wang et al., 2020b). TSG is white amorphous powder with a molecular weight of 406.3833 and a density of 1.593 g/cm^3^. Some physical and chemical properties of TSG are shown in Table 1 (The data in the table are referred to https://baike.baidu.com).

**TABLE 1:** Physical and chemical properties of TSG.

Name	2,3,5,4′-Tetrahydroxystilbene 2-O-glucoside
Alias	(2S,3R,4S,5S,6R)-2-[2,4-dihydroxy-6-[(E)-2-(4-hydroxyphenyl)vinyl]phenoxy]-6-(hydroxymethyl)tetrahydropyran-3,4,5-triol
Source	*Polygonum multiflorum* Thunb.
CAS number	82373-94-2
EINECS number	2017-001-1
Compound type	Glycosides
Molecular formula	C_20_H_22_O_9_
Molecular weight	406.3833
Properties	Powder
Color	White
Solubility	H_2_O: soluble 5 mg/ml, clear (warmed)
Density	1.593 g/cm^3^
pKa	8.87 ± 0.40 (Predicted)
Boiling point	715°C at 760 mmHg
Flash point	386.2°C
Vapour pressure	1.86E-21 mmHg at 25°C
Refractivity	1.76
Polar surface area	160.07000
LogP	0.15250
Storage conditions	2–8°C, dry, dark and sealed

## 3 Pharmacological Properties of TSG

**FIGURE 2:**
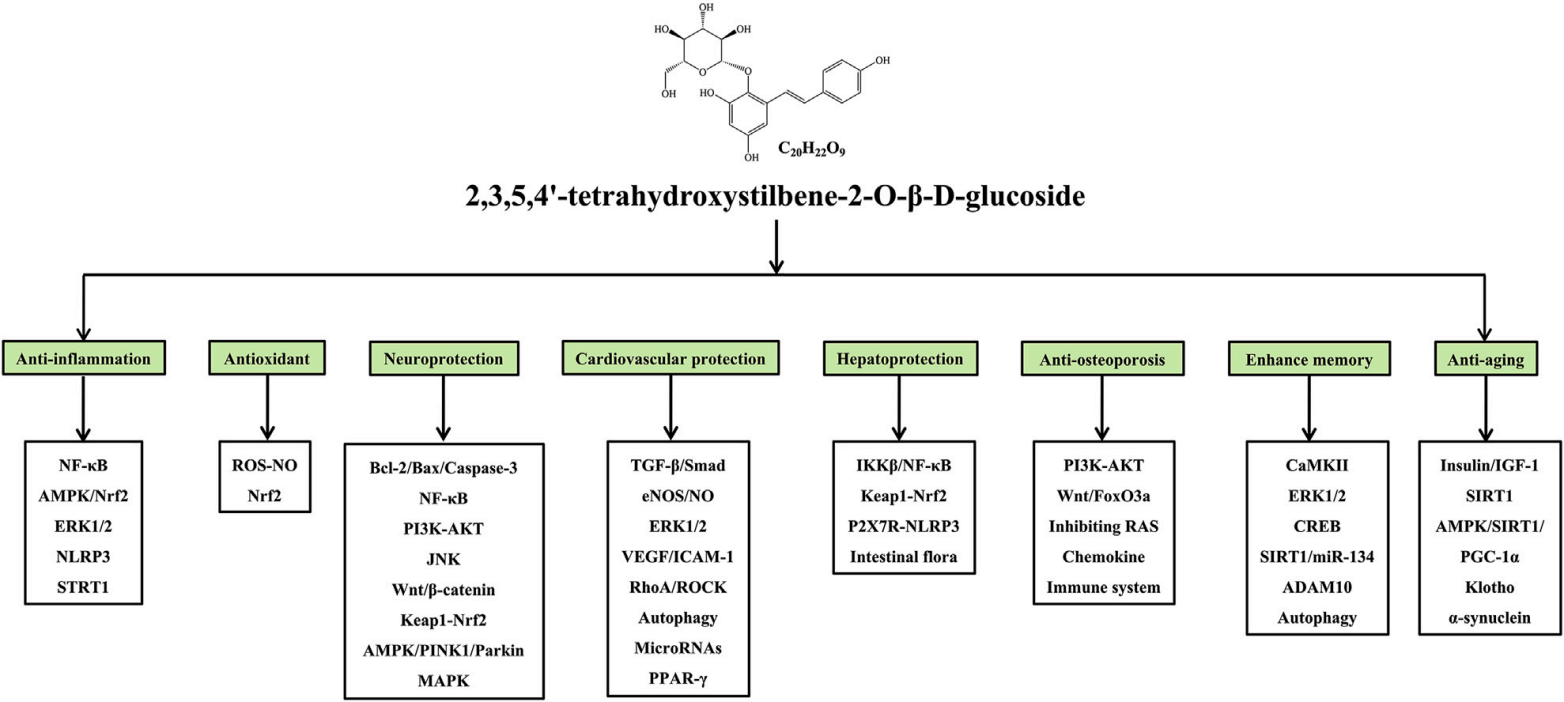
Summary of pharmacological effects of TSG.

**FIGURE 3:**
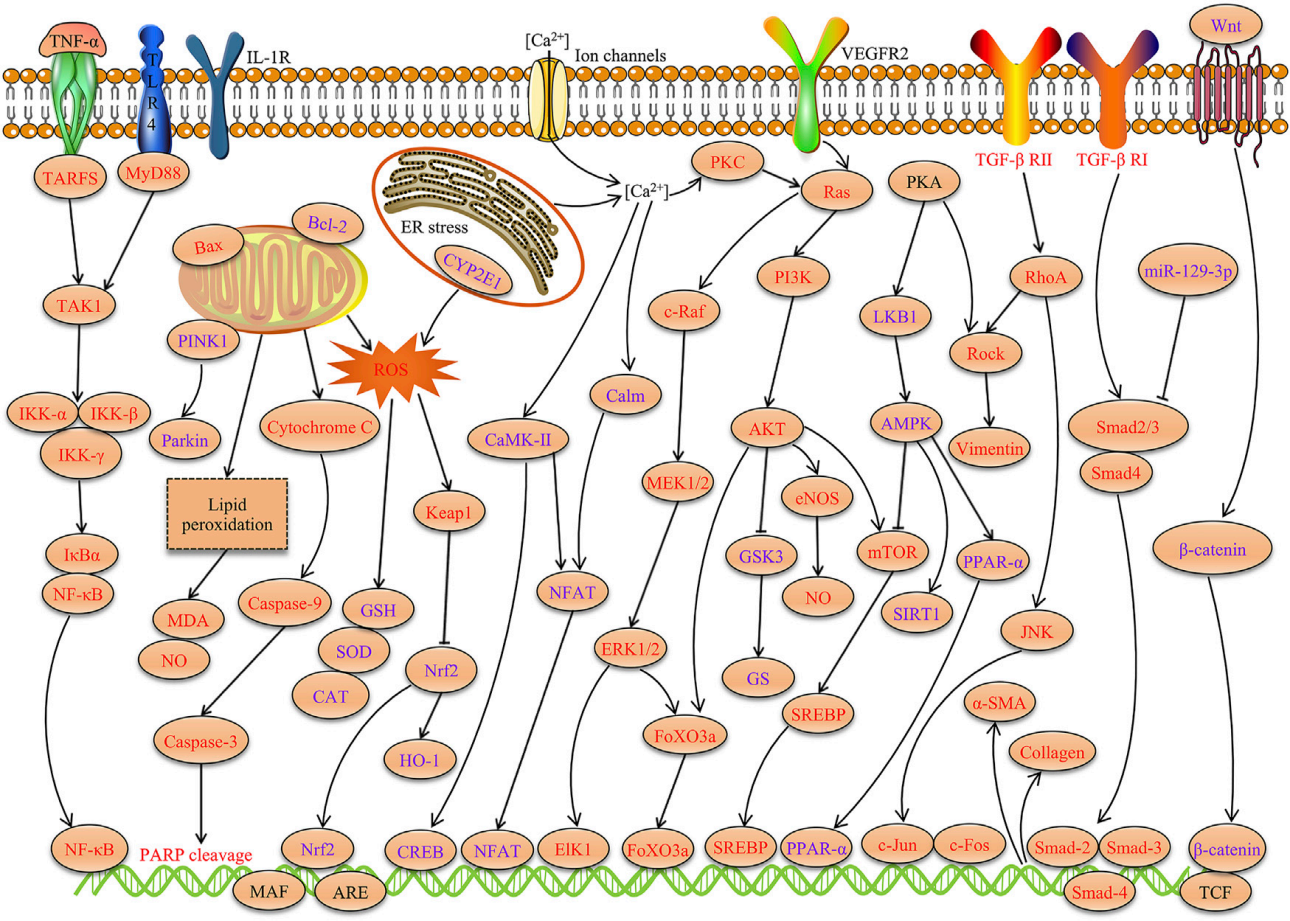
Molecular pathways involved in the pharmacological properties of TSG. TSG exerts significant anti-inflammation, antioxidant, neuroprotection, cardiovascular protection, hepatoprotection, anti-osteoporosis, enhancement of memory and anti-aging activities through modulating these signaling transduction pathways (Red represents the inhibitory effect of TSG and purple represents the promotion effect of TSG).

Many studies have confirmed that TSG has various pharmacological properties through modulating multiple signaling transduction pathways, such as NF-κB, PI3K-AKT, ERK1/2, AMPK, Nrf2, Bcl-2/Bax/Caspase-3, ROS-NO, TGF-β/Smad, MAPK, and SIRT1. The pharmacological properties of TSG are shown in Table 2. The molecular pathways involved in the pharmacological action of TSG are presented in Figure 2 and Figure 3.

**TABLE 2:** Molecular mechanisms to the pharmacological activity of TSG.

Models	Dosage of administration	Molecular mechanisms	References
Anti-inflammatory effects			
Mouse podocytes in high glucose	0.1, 1, 10 μM	Suppression of NLRP3 inflammasome signaling	Li et al. (2018)
Acetic acid-induced acute colitis mice	60, 120 mg/kg	Alleviating oxygen and nitrogen free radicals level and down-regulating iNOS expression	Wang et al. (2008)
Mitomycin C-induced chronic colitis mice	60 mg/kg		
LPS-stimulated microglia	10, 20, 40, 80, 100 μM	Inhibiting NF-κB and activating AMPK/Nrf2 pathways	Park et al. (2016)
Human gingival fibroblasts	10, 25, 100, 200 μM	Activated ERK1/2 and AMPK and inhibited NF-κB activation	Chin et al. (2016)
Ligature-induced rat modeling periodontitis	0.1, 10 mg/kg		
Murine macrophage cell line RAW264.7	120, 240, 480 μM	Enhance mitochondrial biogenesis and function via activation the HO-1	Yu et al. (2017)
Atherosclerotic rats	30, 60, 120 mg/kg	Suppress the expression of MMP-2 and MMP-9 and inhibit inflammation	Zhang et al. (2008)
Acetic acid-induced experimental colitis in mice	10, 30, 60 mg/kg	Upregulation of PPAR-γ and inhibition of the NF-κB inflammatory pathway	Zeng et al. (2011)
Chronic inflammatory pain caused by injection of CFA into the hind paw of mice	50, 100, 200 mg/kg	Repressed p38/NF-κB signaling pathway and reduced the expression of TNF-α	Fan et al. (2018a)
LPS-induced mouse microglia BV2 cells	20, 40, 80 μM	Activation of NADPH oxidase and NF-κB signaling pathways	Zhang et al. (2013a)
Antioxidant effects			
6-OHDA-induced apoptosis in PC12 cells	10, 20, 50 μM	Regulation of ROS-NO pathway	Tao et al. (2011)
Gentamicin-induced apoptosis in mouse cochlear UB/OC-2 cells	5, 10, 20 μM	Suppressing ROS production and downregulating the mitochondrial-dependent apoptotic pathway	Wen et al. (2020)
H_2_O_2_-induced human brain microvascular endothelial cell	50, 10 μM	Inhibiting oxidative stress and inflammatory responses	Jiang et al. (2017)
Hypertensive rats	50 mg/kg	Improvement of Klotho mediated renal injury, antioxidation and bone metabolism	Ling et al. (2016)
Mouse cochlear UB/OC-2 cells	1.25, 2.5, 5, 10, 20, 40 μM	Inhibiting both autophagy and the apoptosis pathway	Wu et al. (2020)
UVB-induced human skin fibroblasts	20, 100, 500 μM	Improving oxidative stress and inhibiting MMP-1 expression	Meijiao and Yifeng (2019)
Neuroprotective effects			
PC12 cells	5, 10 μM	Restoring antioxidant defense system and inhibiting p38 MAPK pathway	Zhang et al. (2017a)
Mitochondrial dysfunction rat model induced by NaN_3_	60 mg/kg	Enhancing mitochondrial function, decreasing Aβ production and increasing neurotrophic factors	Zhang et al. (2018c)
APP/PS1 mice	50, 100 mg/kg	Reduction of Aβ deposits in the brain	Gao et al. (2021)
APP/PS1 transgenic mice	50 mg/kg	Activation of AKT-GSK3β pathway	Yin et al. (2018)
LPS/ATP and Aβ25-35-induced inflammation inmicroglia and neurons	0.01, 0.1, 10, 100 μM	By mitophagy via AMPK related PINK1/Parkin signaling pathway	Gao et al. (2020)
Human dopaminergic neuroblastoma SH-SY5Y cells	3.125, 6.25, 12.5, 25, 50 μM	Improving mitochondrial function and inhibiting apoptosis	Sun et al. (2011)
Ischemic model of oxygen-glucose deprivation followed by reperfusion	15, 40 mg/kg	Involvement of JNK, SIRT1 and NF-κB pathways, and inhibition of intracellular ROS/RNS generation	Wang et al. (2009b)
Ischemic model of middle cerebral artery occlusion	15, 40 mg/kg		
HT22 hippocampal cells	50, 100 μg/ml	Decreasing ROS production and stabilizing MMP	Lee et al. (2017)
MPP^+^-induced apoptosis in PC12 cells	1, 5, 10 μM	Inhibiting ROS generation and modulating JNK activation	Li et al. (2010b)
C57BL/6 mice hippocampal neurons	100 μM	Promote NMDA-mediated EPSC via PKCβ cascade	Tong et al. (2020)
MPP^+^-induced apoptosis in PC12 cells	0.1, 1, 10 μM	Involvement of PI3K/AKT pathway activation	Qin et al. (2011)
Mouse model of PD	20, 40 mg/kg	Through PI3K/AKT signaling pathway	Zhang et al. (2013b)
Timed pregnant BALB/c mice neural stem cells	10 μM	Triggering the Wnt/β-catenin signaling pathway	Zhang and Yang (2021)
Aβ-induced N9 and BV2 cells	90 μM	Regulating PU.1 expression	Jiao et al. (2018)
A53T AS cells plus MPP^+^ exposure	3.125, 6.25, 12.5, 25, 50 μM/L	Inhibiting α-Syn over-expression and aggregation, and enhancing mitochondria function	Zhang et al. (2017b)
6-OHDA-elicited DA neuronal injury	10, 50 mg/kg	Inhibition of microglia-elicited neuroinflammation	Huang et al. (2018)
Primary rat midbrain neuron-glia	10, 50 mg/kg		
Microglia BV2 cell	20, 40, 80 μM	Activation of NADPH oxidase and NF-κB signaling pathways	Zhang et al. (2013a)
MCAO model	3, 6, 12 mg/kg	Suppress the NADPH-induced OS and reduce neuronal apoptosis and autophagy	Yu et al. (2019)
CNS injuries mouse model	100 mg/kg	Improving antioxidant and anti-inflammatory capacity	Zhou et al. (2020)
Mouse primary-cultured astrocytes	80 μM	Increasing the GLT-1 protein expression level by the activation of AKT	Chen et al. (2017)
LPS-induced rat DA neuronal damage	10, 50 mg/kg	Attenuating microglia-mediated neuroinflammation and enhancing astroglia-derived neurotrophic effects	Zhou et al. (2018)
Primary rat midbrain neuron-glia	20, 40, 80 μM		
Aβ_1-42_-induced AD mice	30, 60, 120 mg/kg	Through Keap1/Nrf2 antioxidant pathway	Xie et al. (2018)
Cerebral ischemia reperfusion rats	60, 120 mg/kg	Up-regulating the expression of NCF protein, activating the PKA pathway and increasing the expression of GAP-43 protein, a marker of axonal regeneration	Jie et al. (2010)
Cardiovascular protection effects			
PDGF-BB induced VSMCs proliferation	100 μM	Involvement of NO/cGMP/PKG pathway	Xu et al. (2012)
Ang Ⅱ-induced proliferation of rat VSMCs	10, 50, 100 μM/L	Suppression of the Src-MEK1/2-ERK1/2 signal pathway	Xu et al. (2015)
Sprague-Dawley male rats	30, 60, 120 mg/kg	Suppressing the expression of adhesion (ICAM-1/VCAM-1) molecules	Wang et al. (2013)
HFD-induced mice	35, 70 mg/kg	Improvement of lipid accumulation and inflammation, and regulation of the intestinal microbial imbalance	Li et al. (2020b)
PMA-induced U937 cells	10, 25, 50, 100 mM	Interruption of TGFβ/Smad pathway and Caspase-3 activation	Yao et al. (2016a)
oxLDL-induced U937 cells	10, 25, 50, 100 mM		
Rat carotid artery balloon injury model	50, 100 mg/kg	Through the SCF/c-kit, SDF-1α/CXCR4 and FKN/CX3CR1 axes	Hu et al. (2019)
Pressure overload-induced cardiac fibrosis	120 mg/kg	Up-regualtion of endogenous PPAR-γ expression	Peng et al. (2016)
C57BL/6 male mice	40, 80, 160 mg/kg	Through both endothelium-dependent and endothelium-independent mechanisms	Jia et al. (2019)
Neonate rat cardiomyocytes	30, 100, 300 μM/L	By decreasing ROS generation and intracel-lular [Ca^2+^] and by inhibiting apoptotic signaling pathways	Zhang et al. (2009a)
Acute mouse model of DOX-induced cardiotoxicity	0.1 g/kg		
Rats on the atherosclerotic diet	30, 60, 120 mg/kg	By preventing changes of eNOS and iNOS expression	Zhang et al. (2009b)
Rat heart remodeling model by abdominal aorta banding	30, 60, 120 mg/kg	Inhibition of ERK1/2 and p38 MAPK activation	Xu et al. (2014)
PDGF-BB-induced VSMC proliferation	10, 25, 50 μM/L	Inhibition of ERK1/2 activation and regulation of cell cycle regulator expression	Xu et al. (2011)
Rat VSMCs	1, 10, 25, 50, 100 μM/L	Inhibiting vimentin rearrangement and expression	Xu et al. (2011)
Atherosclerosis in ApoE^−-^ mice	50, 100 mg/kg	Promoting reverse cholesterol transport	Chen et al. (2018b)
HUVECs	1, 10, 25, 50, 100 μM	By inhibiting vimentin expression via the interruption of the TGFβ/Smad signaling pathway	Yao et al. (2015)
HUVECs	1, 10, 25, 50, 100 μM	Regulating vimentin cytoskeleton and its colocalization with ICAM-1 and VCAM-1	Yao et al. (2014)
PA-induced inflammation and apoptosis in cardiomyocytes	0.2, 0.4, 0.8 mM	Regulating miR-129-3p/Smad3 signaling	Zou and Kong (2019)
Macrophage-derived foam cell in hypercholesterolemic NZW rabbits	25, 50, 100 mg/kg	Inhibition of the expression of ICAM-1 and VEGF in foam cells	Yang et al. (2005)
LPS-induced endothelial dysfunction of HUVECs	10, 25, 50, 100 μM	Inhibiting RhoA/ROCK signaling and F-actin remodeling	Qi et al. (2020)
Spontaneously hypertensive male rats	100 mg/kg	Through activating the AKT/mTOR pathway	Dong et al. (2016)
Atherosclerotic rats	120 mg/kg	Altering the expression of Calreticulin, vimentin, HSP 70, lipocortin 1, and Apo A-I	Yao et al. (2013)
Hepatoprotective effects			
HFD-induced NAFLD zebrafish	25, 50, 100 μg/ml	Regulating lipid metabolism related pathway, IKKβ/NF-κB pathway and Keap1-Nrf2 pathway	Wang et al. (2020a)
Ethanol-induced AFLD mice	50, 100 mg/kg	By targeting the crosstalk between macrophages and hepatocytes	Zhang et al. (2020)
HFD-induced NAFLD rats	12, 24, 48 mg/kg	Modulation of the gut microbiota and TLR4/NF-κB pathway	Lin et al. (2015b)
HFD-induced NASH mice	50, 100 mg/kg	Regulating key regulators of lipid metabolism, inflammation, fibrosis and oxidative stress	Xu et al. (2019)
Anti-osteoporosis effects			
MC3T3-E1 mouse osteoblast precursor cells	1, 10 μM/L	Through Wnt/FoxO3a signaling, the immune system and the chemokine signaling pathway	Zhang et al. (2018a)
Murine pre-osteoblastic MC3T3-E1 cells	0.01, 0.1, 1 μg/ml	Through PI3K/AKT pathway	Fan et al. (2018b)
Rat mesenchymal stem cells	6.25, 12.5, 25 μg/ml	Promoting the alkaline phosphatase activity and increasing the osteocalcin content, and increasing the nodule area	Zheng et al. (2017)
DEX-induced zebrafish osteoporosis model	3.12, 6.25, 12.50 μg/ml		
Diabetic mouse model induced by streptozotocin	10, 40 mg/kg	Regulation on osteogenesis and osteoclastogenesis	Zhang et al. (2019)
Memory-enhancing effects			
C57BL6J mice	20, 40, 80 mg/kg	Promoting hippocampal memory and synaptic plasticity, and activating ERKs, CaMKII and SIRT1/miR-134	Chen et al. (2016)
Sprague-Dawley rats	30, 60 mg/kg	Protecting neuronal synaptic structures, and enhancing SYP expression	Wang et al. (2007)
Sprague-Dawley male rats	50 mg/kg	Through the APP pathway	Hou et al. (2011)
APP695V717I transgenic mouse models	100 mg/kg	Decreasing the expression of Beclin-1 and LC3-II in autophagy pathways	Luo et al. (2015)
AD-like APP transgenic mice	120, 240 μM/kg	Antagonizing the effects of amyloid plaques or inhibiting the formation of amyloid plaques directly	Zhang et al. (2006)
Hippocampal synaptic in normal mice	1, 5 μM	Increasing the intracellular calcium level and facilitating hippocampal long-term potentiation	Wang et al. (2011)
Sprague-Dawley male rats	50 mg/kg	Protecting synaptic structure and function	Zhou et al. (2012)
Alzheimer’s disease-like mouse model	120, 240 μM/kg	Prevent over-expression of α-synuclein and reverse the increased expression of α-synuclein	Zhang et al. (2013c)
Anti-aging effects			
PMBMECs from the SAMP8	10, 20, 50 μM	Upregulating neural klotho and downregulating neural insulin or insulin-like growth factor 1	Zhou et al. (2015)
SAMP8	2, 20, 50 μM		
Ang II-induced HUVECs	20, 40 μg/ml	Modulation of SIRT1 activity	Fan et al. (2021)
Aged C57BL/6 female mice	40.6 mg/kg	Regulation of the AMPK/SIRT1/PGC-1α signaling cascade	Ning et al. (2018)
Mouse myoblast cell line C2C12	25, 50, 100 μM		
Human cervical cancer cell	50, 100, 200 μM		
Specific pathogen free C57BL/6J female mice	50, 100, 200 mg/kg	Through protecting synapses and inhibiting α-synuclein overexpression and aggregation in multiple brain regions	Shen et al. (2015)
Dermas on skin of ageing mice	180 mg/kg	Inhibition of insulin/IGF-1 signaling pathway	Zhou et al. (2014)
Other pharmacological effects			
Male Sprague-Dawley rats	10, 20 mg/kg	Involvement of SIRT1 and TGF-β1 pathway	Li et al. (2010a)
Cultured rat mesangial cells	1, 5, 25, 100 μM		
C57BL/6J male mice	30, 60 mg/kg	Enhancement of the hippocampal BDNF system	Wang et al. (2017)
Human dental pulp stem cells	0.1, 1, 10, 100 μM	Through AMPK/ERK/SIRT1 axis	Lin et al. (2018)
C57BL/6J mice	200 μM	Inhibition of p53, Fas, and Bax induced apoptosis	Chen et al. (2018a)
Kunming mice	10, 20, 40, 80 mg/kg	Regulating sleep duration via LDH and salivary alpha amylase	Wei et al. (2017)
Traumatic brain injury mice model	12 mg/kg	Inhibition of the Ras/JNK signaling pathway	Cao et al. (2020)

### 3.1 Anti-Inflammatory Effects

Inflammation is a defensive response to various harmful physiological stimuli (such as infection, tissue injury, tissue stress, and dysfunction), which can activate various immune cells and tissues, including macrophages, lymphocytes, monocytes, and neutrophils (Medzhitov, 2008). Inflammation is not only an important component of the immune system, but also the basis of various physiological and pathological processes, which is closely associated with the occurrence of many chronic diseases, such as cardiovascular disease, neurodegeneration, inflammatory bowel disease, diabetes, and cancer (Yeung et al., 2018). A study has shown that NO and prostaglandin E2 (PGE2), controlled by the expression of inducible nitric oxide synthase (iNOS), endothelial nitric oxide synthase (eNOS) and cyclooxygenase-2 (COX-2), are responsible for the development of inflammation (Yun et al., 2008). In addition, lipopolysaccharide (LPS), a bacterial endotoxin, can activate endothelial cells and promote the release of a large number of inflammatory cytokines, ultimately leading to the activation of inflammatory signaling cascade and immune dysfunction (Dauphinee and Karsan, 2006; Shi et al., 2014). Interestingly, a large number of recent experimental studies have reported the therapeutic effect of TSG on various inflammatory models.

In LPS-induced RAW264.7 cells, TSG (120, 240, 480 μM) inhibited macrophage activation and inflammation by attenuating the secretion of pro-inflammatory cytokines, including interleukin-6 (IL-6) and tumor necrosis factor-α (TNF-α) (Yu et al., 2017). Similarly, in high glucose-induced podocytes of mice, TSG (0.1, 1, 10 μM) protected podocytes from apoptosis by decreasing NLRP3 inflammasome and interleukin-1β (IL-1β) expression (Li et al., 2018). Moreover, in a *Porphyromonas gingivalis*-induced inflammation model in human gingival fibroblasts and a ligature-induced periodontitis model in rats, TSG showed anti-inflammatory effects by enhancing the AMPK activation and sirtuin 1(SIRT1) expression to suppress the production of inflammatory cytokines, such as TNF-α, IL-1β and IL-6, which was attributed to the activation of extracellular signal-regulated kinase 1/2 (ERK1/2) and AMPK as well as the inactivation of the NF-κB signaling pathway (Chin et al., 2016). Taken together, TSG may serve as a potential drug for the treatment of inflammatory diseases.

In acetic acid-induced acute colitis in mice, TSG (60, 120 mg/kg) significantly ameliorated the colonic injury by reducing the levels of myeloperoxidase (MPO), malonaldehyde (MDA) and NO as well as increasing superoxide dismutase (SOD) activity (Wang et al., 2008). Similarly, in mitomycin C-induced chronic colitis in mice, TSG (60 mg/kg) significantly ameliorated the colonic injury by modulating the above indicators (Wang et al., 2008). The beneficial effects of TSG on acute and chronic experimental colitis are mainly attributed to attenuating oxygen and nitrogen radical levels and down-regulating iNOS expression (Wang et al., 2008). Interestingly, the study of Zeng et al. (2011) also confirmed the protective effects of TSG (10, 30, 60 mg/kg) against acetic acid-induced experimental colitis in mice. Specifically, TSG significantly attenuated acetic acid-induced colonic injury, such as reversing weight loss and ameliorating histopathological changes (Zeng et al., 2011). In addition, TSG can decrease the expression of the inflammatory mediators such as TNF-α, IL-6 and cyclooxygenase-2 (COX-2) as well as the MDA content. The protective mechanisms mainly involved the up-regulation of peroxisome proliferator-activated receptor γ (PPAR-γ) and the inhibition of NF-κB pathway (Zeng et al., 2011). These suggest that TSG may be a promising candidate for the treatment of inflammatory bowel diseases.

In recent years, neuroinflammation has been increasingly implicated in the pathogenesis of neurological diseases, including trauma, stroke and neurodegenerative disorders (González et al., 2014). Neuroglia cells are considered to be the marker of neuroinflammation, particularly the activation of microglia (Voet et al., 2019). Once activated, microglia produce large amounts of proinflammatory cytokines, such as IL-1β, TNF-α, NO and reactive oxygen species (ROS), thus leading to neuronal damage (Voet et al., 2019; Shields et al., 2020). Therefore, inhibiting the activation of microglia may have an ameliorative effect on neuroinflammation-associated diseases. An earlier study showed that TSG (20, 40, 80 μM) inhibited microglia activation by reducing LPS-induced release of proinflammatory factors such as TNF-α, IL-1β and NO in microglia, as well as attenuating LPS-induced NADPH oxidase activation and ROS generation, which further attenuated neuroinflammation (Zhang et al., 2013a). The anti-inflammatory effect was associated with the diminished activation of NADPH oxidase and NF-κB signaling pathway (Zhang et al., 2013a). Similarly, the study by Park et al. (2016) showed that TSG (10, 20, 40 μM) could inhibit the NF-κB and activate the AMPK/Nrf2 pathway to attenuate LPS-induced neuroinflammatory responses in microglia, thereby exerting an anti-inflammatory effect. In addition, Fan et al. (2018a) showed that TSG (50, 100, 200 mg/kg) also alleviated chronic inflammatory pain induced by complete Freund’s adjuvant in mice by inhibiting the neuronal apoptosis, microglial activation, and GluN2B overexpression in the anterior cingulate cortex. The protective effect is mainly attributed to the inhibition of p38/NF-κB signaling pathway (Fan et al., 2018a). Therefore, TSG is expected to be a new anti-inflammatory and analgesic drug.

### 3.2 Antioxidant Effects

Oxidative stress refers to the excessive production of highly reactive molecules such as ROS and reactive nitrogen, and the degree of oxidation exceeds the ability of cells to scavenge oxides, resulting in the disruption of redox signaling and control, and/or molecular damage (Sies et al., 2017). As we all know, the oxidation reaction of ROS is considered to be a key factor in triggering oxidative stress (Kuwabara et al., 2008). When ROS accumulates excessively in the body, it will produce serious cytotoxicity because of its damage on DNA, protein, and other macromolecules as well as lipid peroxidation (Dixon and Stockwell, 2014). At present, oxidative stress is considered as an important factor of aging and age-related diseases, such as type 2 diabetes, cardiovascular disease, neurodegenerative disease, and cancer (Valko et al., 2006; Luo et al., 2020). The indicators of the damage caused by oxidative free radicals mainly include antioxidant enzymes, such as SOD, catalase (CAT), glutathione peroxidase (GSH-Px), and lipid peroxidation products, such as MDA, 4-hydroxynonenoic acid (Sehirli et al., 2008). Interestingly, a large amount of literature showed that TSG had antioxidant activity by regulating the expression of the oxidative damage markers, which suggested that TSG might be an effective antioxidant.

In human dermal fibroblasts, TSG (20, 100, 500 μM) showed protective effects on UV-B-induced stress premature senescence by up-regulating SOD and inhibiting MDA (Meijiao and Yifeng, 2019). Similarly, in human brain microvascular endothelial cells, TSG (50, 100 μM) exhibited protective effects on hydrogen peroxide-caused oxidative stress by increasing the activity of SOD and GSH, as well as reducing the content of MDA and ROS (Jiang et al., 2017). Furthermore, in 6-hydroxydopamine-induced rat adrenal pheochromocytoma PC12 cells, TSG (10, 20, 50 μM) showed antioxidant effects by inhibiting intracellular ROS and NO overproduction and decreasing apoptotic cells in a dose-dependent manner, which was attributed to the regulation of the ROS-NO signaling pathway (Tao et al., 2011). These indicate that TSG can provide effective protection for oxidative stress-related diseases.

In addition, in gentamicin-induced mouse cochlear UB/OC-2 cells, TSG (5, 10, 20 μM) inhibited the ototoxicity by decreasing the ROS production and activating the antioxidant enzyme SOD (Wen et al., 2020). Nuclear factor erythroid 2-related factor 2 (Nrf2) is a key transcription factor in the cellular anti-oxidative stress system (Vomhof-Dekrey and Picklo, 2012). The Nrf2 signaling pathway regulates oxidative stress mainly by regulating the transcription of various detoxification genes, which plays an important role in the mechanism of resisting endogenous or exogenous oxidative stress (Bellezza et al., 2018). When the redox balance is destroyed, Nrf2 enters the nucleus and activates the transcription of a variety of antioxidant genes, reducing the cell damage caused by ROS and electrophilic substances, which maintains the physiological balance of oxidation-antioxidation in the body (Stępkowski and Kruszewski, 2011; Bellezza et al., 2018). In H_2_O_2_-induced oxidative stress of UB/OC-2 cells, TSG (5, 10, 20, 40 μM) showed a strong ability of antioxidant stress by increasing the level of antioxidant/detoxification enzymes such as heme oxygenase 1 (HO-1), NQO1, glutathione-s-transferase (GST), and UGT, as well as inhibiting the excessive production of ROS, apoptosis, and autophagy, which was attributed to the activation of the Nrf2 pathway (Wu et al., 2020). These results suggest that TSG-mediated activation of the Nrf2 signaling pathway may be an important mechanism of enhancing endogenous antioxidant defense system.

### 3.3 Neuroprotective Effects

Currently, inflammatory response and oxidative stress are well recognized by the medical community as key events during the development of neurodegenerative diseases (Amor et al., 2014; Singh et al., 2019). Fortunately, in recent years, there have been a great deal of literature reporting that TSG shows neuroprotective effects in several neurodegenerative diseases, such as Alzheimer’s disease (AD), Parkinson’s disease (PD), and cerebral ischemic injury, specifically manifested by inhibiting microglia-mediated neuroinflammation, enhancing mitochondrial function, decreasing oxidative stress, inhibiting neuronal apoptosis, reducing α-synuclein and amyloid β-protein (Aβ) production, and increasing neurotrophic factors (Zhang et al., 2013b; Xie et al., 2018; Yu et al., 2019). TSG may exert neuroprotective effects through the following molecular mechanisms and show synergistic biological effects through their anti-inflammatory and antioxidant activities.

#### 3.3.1 Bcl-2/Bax/Caspase-3

In 1-methyl-4-phenylpyridinium (MPP^+^)- or glutamate-induced cytotoxicity or apoptosis models, TSG significantly reduced the markers of oxidative stress and exerted neuroprotective effects through multiple pathways due to its excellent antioxidant activity (Zhang et al., 2017a; Lee et al., 2017). On the one hand, TSG significantly enhances the activities of antioxidant enzymes in cells, including SOD, CAT and GSH-Px, as well as decreases MDA content (Sun et al., 2011; Zhang et al., 2017a). On the other hand, TSG significantly up-regulates the Bcl-2/Bax ratio, reverses cytochrome c release, and inhibits Caspase-3 activation (Zhang et al., 2017b; Lee et al., 2017). In addition, TSG also significantly inhibits the activation of the p38 mitogen-activated protein kinase (MAPK) signaling pathway, whereas ERK phosphorylation is unaffected (Zhang et al., 2017a). Taken together, the mechanisms of the neuroprotective effect of TSG may be related to ameliorating mitochondrial dysfunction, reducing intracellular oxidative stress, and ultimately inhibiting apoptosis by regulating the Bcl-2/Bax/Caspase-3 signaling pathway (Sun et al., 2011; Zhang et al., 2017a; Lee et al., 2017).

α-synuclein, a soluble protein expressed in the presynaptic and perinuclear of central nervous system (CNS), is critically involved in the pathogenesis of PD and the associated dysfunction (Ozansoy and Başak, 2013). The study of Zhang et al. (2017b) showed that TSG inhibited the overexpression and aggregation of α-synuclein to achieve neuroprotection. In addition, in the middle cerebral artery occlusion (MCAO) model, TSG attenuated cerebral ischemia/reperfusion injury and alleviated neurological deficit symptoms by decreasing neurological scores, reducing cerebral infarct volume, ameliorating neuronal damage in the ischemic cortex and hippocampus, and inhibiting the expression of NOX4, activated Caspase-3 (9), and Beclin 1 proteins (Yu et al., 2019). Its protective mechanism may involve inhibiting oxidative stress, and reducing neuronal apoptosis and autophagy (Yu et al., 2019).

#### 3.3.2 Inflammatory Signaling Pathway

Neuroinflammation is considered to be a key event in the development of neurodegenerative diseases, and is mainly characterized by microglia activation (Baune, 2015; Spagnuolo et al., 2018). Therefore, the development of drugs that can antagonize microglial activation and reduce the release of pro-inflammatory cytokines becomes a potentially important target for the treatment of neurodegenerative diseases. One study showed that TSG (20, 40, 80 μM) reduced LPS-induced release of proinflammatory factors (IL-1β, TNFα and NO) in microglia, the activation of NADPH oxidase, and ROS generation (Zhang et al., 2013a). The further study suggested that the neuroprotective effect of TSG may involve the NF-κB signaling pathway activation (Zhang et al., 2013a). Similarly, TSG (10, 50 mg/kg) significantly attenuated dopamine neuron loss in the substantia nigra in a model of rats with LPS-induced dopamine neuron injury (Zhou et al., 2018). Specifically, TSG can double-regulate glial cells by ameliorating microglia-mediated neuroinflammation and enhancing the secretion of astrocyte-derived neurotrophic factors, thus protecting dopamine neurons from LPS-induced neurotoxicity (Zhou et al., 2018).

Aβ deposition is regarded as a key pathogenic event in the progression of AD, so the inhibition of Aβ-induced microglial activation is considered to be an effective strategy for the treatment of AD (Borderud et al., 2014). In Aβ-induced microglial model, TSG attenuated Aβ-induced microglial activation and inflammation, and allowed the differentiation of microglia toward the M2 phenotype (Jiao et al., 2018). Specifically, TSG could significantly inhibit the production of inflammatory molecules, including iNOS, NO, COX-2, and PGE2, as well as increase the levels of M2 markers, including IL-10, brain-derived neurotrophic factors, glial cell line derived neurotrophic factors, and arginase-1 (Jiao et al., 2018). Furthermore, TSG (10, 50 mg/kg) significantly protected dopamine neurons from 6-hydroxydopamine-(6-OHDA)-induced neurotoxicity in a dopamine neuron injury model (Huang et al., 2018). Similarly, in a co-culture model of primary glial cells, TSG (20, 40, 80 μM) also showed similar neuroprotective effects (Huang et al., 2018). A subsequent study showed that TSG-mediated neuroprotection was closely related to the inhibition of microglia activation, the subsequent release of pro-inflammatory factors, and the inactivation of MAPK signaling pathway (Huang et al., 2018). Taken together, the neuroprotective effect of TSG is associated with the regulation of microglia, which is mediated by NF-κB and MAPK signaling pathways.

#### 3.3.3 PI3K-AKT

A large number of recent studies have reported that the PI3K/AKT pathway ensures an active state of the neural defense system, thereby exerting a neuroprotective effect by preventing apoptosis and neuroinflammation (Nakano et al., 2017). Therefore, the search and development of drugs that can modulate the PI3K/AKT pathway is crucial for the prevention and treatment of neurodegenerative diseases. A study showed that TSG (0.1, 1, 10 μM) showed significant neuroprotective effects against MPP^+^-induced PC12 cell injury and apoptosis (Yin et al., 2018). The effect may be partially mediated by the PI3K/AKT signaling pathway (Yin et al., 2018). Two years apart, the team demonstrated the potential neuroprotective effects of TSG (20, 40 mg/kg) in a mouse model of PD (Zhang et al., 2013b). The results showed that TSG could promote the survival of dopamine neurons *in vivo*, and also reconfirmed that TSG-induced neuroprotection was mediated by the PI3K/AKT signaling pathway (Zhang et al., 2013b).

Studies have reported that Glutamate transporter-1 (GLT-1) deficiency has an important impact on neuronal damage in excitotoxicity-related diseases, such as cerebral ischemia and AD (Mookherjee et al., 2011; Sun et al., 2014). Therefore, the drugs which can enhance GLT-1 protein expression may have beneficial effects on neuronal toxicity-associated diseases. Given that TSG had good neuroprotective effects in a series of experimental models, Chen et al. (2017) evaluated the effects of TSG on GLT-1 protein expression in primary astrocytes of mice. Their results showed that TSG (10, 30, 80 μM) significantly increased GLT-1 protein expression in a dose-dependent manner (Chen et al., 2017). More importantly, they found that the increase of GLT-1 protein levels was achieved by the activation of AKT but not ERK1/2 (Chen et al., 2017). Moreover, Yin et al. (2018) also confirmed the neuroprotective effect of TSG on amyloid precursor protein expression by *in vivo* (APP/PS1 mice) and *in vitro* (HEK-293FT cells and SH-SY5Y cells) experiments. The specific mechanisms may be activating the AKT-GSK3β signaling pathway and subsequently attenuating the splicing activity of alternative splicing factors to reduce Aβ deposition (Yin et al., 2018).

#### 3.3.4 JNK

Recently, it has been shown that the Jun N-terminal kinase (JNK) signaling pathway is involved in the occurrence and development of several neuronal diseases, such as cerebral ischemia, intracerebral hemorrhage, AD and PD (Graczyk, 2013). Therefore, the current medical community is trying to develop chemical inhibitors of this pathway to treat neurodegenerative diseases. A previous study showed that TSG (25, 50 μM) could reverse neuronal injury, elevation of intracellular ROS, and dissipation of mitochondrial membrane potential induced by oxygen-glucose deprivation followed by reperfusion in an *in vitro* ischemia model (Wang et al., 2009b). In an *in vivo* cerebral ischemia injury model of mice, TSG (15, 40 mg/kg) could significantly reduce the cerebral infarct volume and the number of positive cells in the cerebral cortex (Wang et al., 2009b). With more in-depth studies, we found that the protective effects of TSG against cerebral ischemia/reperfusion injury mainly involved JNK, SIRT1, and NF-κB pathway (Wang et al., 2009b). Similarly, Li et al. (2010b) showed that in the MPP^+^-induced apoptosis model of PC12 cells, TSG (1, 5, 10 μM) exerted a neuroprotective effect by inhibiting ROS production and regulating JNK activation.

#### 3.3.5 Wnt/β-Catenin

In recent years, cell replacement therapy has been considered as an alternative option for the treatment of neurodegenerative diseases of the CNS (Zhu et al., 2016). In addition, it has been reported that regulating Wnt/β-catenin signaling may improve the cell replacement therapy for PD (L’Episcopo et al., 2014). Interestingly, a recent study confirmed that TSG had a promoting effect on the differentiation of mesencephalic neural stem cells into dopaminergic neurons (Zhang and Yang, 2021). TSG can not only increase the proportion of tyrosine hydroxylase-positive cells and dopamine transporter-positive neurons, the late markers of mature dopaminergic neurons, but also enhance the expression of nuclear receptor-associated factor 1, a specific transcription factor for the development and maintenance of midbrain dopaminergic neurons (Zhang and Yang, 2021). In addition, TSG can also up-regulate the expression of Wnt/β-catenin signaling molecules, including Wnt1, Wnt3a, Wnt5a, and β-catenin (Zhang and Yang, 2021). These findings suggest that TSG can promote neuronal differentiation of neural stem cells in mice by up-regulating the Wnt/β-catenin signaling (Zhang and Yang, 2021). These also suggest that TSG may contribute to the PD treatment by neural stem cell transplantation.

#### 3.3.6 Keap1-Nrf2

Although the etiology and pathogenesis of AD are not fully understood at present in the medical community, a large body of literature has suggested that oxidative stress is an important component of AD’s pathological processes (Wang et al., 2014; Tönnies and Trushina, 2017). In brief, oxidative stress participates in the initiation and progression of AD by promoting Aβ deposition, tau hyperphosphorylation, synaptic dysfunction, and neuronal loss (Chen and Zhong, 2014). Therefore, given the relationship between oxidative stress and AD, antioxidants may be helpful in AD treatment. A study showed that TSG (30, 60, 120 mg/kg) reversed Aβ_1-42_-induced alterations of cognitive behavior, biochemical changes, and oxidative damage in mice. Specifically, TSG significantly reduced MDA and GSSG levels, and increased GSH, CAT, and SOD activities in the hippocampus and cortex (Xie et al., 2018). In addition, TSG also increases Nrf2 and HO-1 protein expression as well as decreases Keap1 protein expression in a dose-dependent manner (Xie et al., 2018). These beneficial effects of TSG are mainly attributed to the inhibition of Keap1/Nrf2 pathway in hippocampal and cerebral cortical tissues (Xie et al., 2018). Meanwhile, it also suggests us that TSG can be used as a natural agent for the treatment of AD.

#### 3.3.7 AMPK/PINK1/Parkin

AMPK is generally activated upon a decline in energy supply and is closely associated with autophagy especially mitophagy (Hang et al., 2019). The expression of PTEN-induced putative kinase 1 (PINK1) on the outer membrane of dysfunctional mitochondria is promoted by mitophagy, and synchronously elevates Parkin, an E3 ubiquitin ligase (Devi et al., 2017). This suggests that the AMPK/PINK1/Parkin pathway may be a new and attractive target for the treatment of AD. Gao et al. (2020) showed that TSG (0.1, 1, 10 μM) had neuroprotective effects on LPS/ATP- and Aβ_25-35_-induced microglial and neuronal inflammation. Specifically, TSG treatment significantly reduced inflammatory cytokine secretion and the NLRP3 inflammasome activation, and regulated mitophagy (Gao et al., 2020). More importantly, the team also found that the protective effect of TSG was abolished when PINK1 or parkin was knocked down by the siRNA or the CRISPR/Cas9 system (Gao et al., 2020). These results suggest that the neuroprotective effects of TSG may be achieved by enhancing AMPK/PINK1/Parkin-dependent mitophagy to attenuate inflammatory damage (Gao et al., 2020).

#### 3.3.8 Others

In cerebral ischemic injury, nerve growth factor (NGF) plays an important role on reducing neuronal injury, improving survival rate, and repairing injury (Allen et al., 2013). Therefore, it is of great significance for the protection of neuronal injury after cerebral ischemia to adopt therapeutic measures that can continuously promote NGF expression. A study showed that TSG (60, 120 mg/kg) had a certain neuroprotective effect on rats with cerebral ischemia-reperfusion (Jie et al., 2010). According to the experimental results of biochemical pharmacology, the neuroprotective mechanisms mainly involved up-regulating the NCF protein expression, activating the PKA pathway, and increasing the growth associated protein-43 (GAP-43) expression, a marker of axon regeneration (Jie et al., 2010). In addition, in the rat model with sodium azide-induced mitochondrial dysfunction, TSG (60, 120 mg/kg) was proved to slow the AD progression (Zhang et al., 2018c). The mechanisms mainly included increasing mitochondrial COX activity, reducing Aβ expression by inhibiting the production of amyloidogenic β-amyloid precursor protein (APP), β-site APP cleaving enzyme 1 (BACE1) and presenilin 1 (PS1), and increasing the expression of neurotrophic factors including NGF, brain-derived neurotrophic factor (BDNF), and its receptor tropomyosin-related kinase B (TrkB) (Zhang et al., 2018c). The latest research showed that long-term TSG (50, 100 mg/kg) treatment significantly improved the cognitive impairment by reducing the deposition of Aβ plaques in the hippocampus and cortex in the APP/PS1 AD mouse model, thereby preventing AD (Gao et al., 2021). In summary, these findings raise the possibility that TSG becomes a new drug for the treatment of neurodegenerative diseases such as AD.

### 3.4 Cardiovascular Protection Effects

The morbidity and mortality of cardiovascular diseases are increasing year by year globally, which have been a major public health problem (Feng et al., 2016). According to epidemiological statistics, approximately 17.8 million deaths due to cardiovascular diseases occurred worldwide in 2017 (Jagannathan et al., 2019). There is clear evidence that some medicines can improve the clinical prognosis of patients with stable cardiovascular diseases, including statins, aspirin, β-receptor blockers and ACE-I/angiotensin receptor blockers, which are even better than surgical treatment (Leong et al., 2017). However, therapeutic affordability and accessibility remain major challenges for cardiovascular disease treatment. Therefore, there is no delay to develop safe, effective, and economical drugs for the treatment of cardiovascular diseases. Fortunately, a large number of studies have shown that TSG is a potential therapeutic agent for cardiovascular diseases, mainly in hypolipidemia, protecting endothelial cells, anti-atherosclerosis, inhibiting vascular smooth muscle cell (VSMC) proliferation, and protecting the myocardium (Xu et al., 2011; Yao et al., 2013; Peng et al., 2016; Chen et al., 2018b; Qi et al., 2020). The signaling pathways involved in treating cardiovascular diseases by TSG are summarized as follows.

#### 3.4.1 TGF-β/Smad

TGF-β/Smad signaling pathway is closely related to cardiovascular diseases, and plays an important role in the pathogenesis of hypertension, atherosclerosis, coronary heart disease, heart failure, myocardial infarction, and other diseases (Zeng et al., 2016). A study showed that TSG (50, 100 µM) protected human umbilical vein endothelial cells (HUVECs) from oxidized low-density lipoprotein (oxLDL)-induced endothelial dysfunction by inhibiting the expression and cleavage of vimentin, and the expression of adhesion molecules and their co-localization with vimentin (Yao et al., 2014). The protective effect may be closely related to blocking the TGF-β/Smad signaling pathway and activating Caspase-3 (Yao et al., 2014). Subsequently, in the model of TNF-α-treated HUVECs, the research team again confirmed that TSG (50, 100 µM) could inhibit the vimentin expression by blocking the TGF-β/Smad signaling pathway, thereby protecting HUVECs from TNF-α-induced cell damage (Yao et al., 2015).

Macrophage-derived foam cell formation is an important event in the development of atherosclerosis (Chistiakov et al., 2017). In the U937 cell model induced by phorbol-12-myristate-13-acetate (PMA) and oxLDL, TSG (50, 100 µM) treatment significantly inhibited PMA-induced cell differentiation, and oxLDL-induced macrophage apoptosis and foam cell formation (Yao et al., 2016a). More importantly, TSG also attenuated PMA- and OXLDL-induced Caspase-3 activation and adhesion molecule levels (Yao et al., 2016a). The specific mechanisms may be interrupting TGF-β1/Smad signaling and Caspase-3 activation to inhibit the expression and degradation of vimentin (Yao et al., 2016a). In addition, increased proliferation and migration of vascular smooth muscle cells (VSMC) is also a key step in the formation of atherosclerotic lesions (Grootaert et al., 2018). Yao et al. (2016b) showed that TSG (50, 100 µM) significantly inhibited TNF-α-induced migration of VSMCs. At the same time, it could also inhibit the expression of TGF-β1 and TGF-βR1, the phosphorylation of TGF-βR1 and Smad2/3, and the nuclear translocation of Smad4 (Yao et al., 2016b). The effect of TSG may be achieved by blocking the TGF-β/Smad signaling pathway to inhibit the redistribution and expression of vimentin.

#### 3.4.2 eNOS/NO

Abnormal metabolism of NO has been found to be a predisposing factor for a variety of cardiovascular diseases (Bryan, 2018). Moreover, endothelial function is mainly based on the function and activity of eNOS, a rate limiting enzyme of NO synthesis (Daiber et al., 2019). Therefore, eNOS/NO signaling pathway has an important regulatory effect on cardiovascular function. A study showed that TSG (30, 60, 120 mg/kg) attenuated intimal hyperplasia and improved endothelial function in rats with atherosclerosis (Zhang et al., 2009b). The molecular mechanisms might be related to preventing the changes of eNOS and iNOS gene and protein expression as well as the consequent increased NO production (Zhang et al., 2009b). It is well known that VSMC proliferation is a critical step in the development of atherosclerosis, which is closely associated with other cellular processes such as inflammation, apoptosis, and matrix alterations (Bennett et al., 2016). Therefore, the inhibition of VSMC proliferation may have beneficial effects on atherosclerosis. Two years apart, the team confirmed again that TSG (100 μM) had an inhibitory effect on platelet derived growth factor (PDGF)-BB-stimulated VSMC proliferation (Xu et al., 2012). The molecular mechanisms may involve the NO/cGMP/PKG signaling pathway (Xu et al., 2012).

#### 3.4.3 ERK1/2

ERK1/2 are involved in the Ras/Raf/MEK/ERK signal transduction cascade, which is involved in the regulation of several processes, including cell adhesion, cell cycle progression, cell survival, cell proliferation, cell differentiation, gene transcription, and so on (Wortzel and Seger, 2011; Roskoski, 2012). Therefore, ERK1/2 play an important role in the pathogenesis and pathophysiology of several diseases. Xu et al. (2011) showed that TSG (10, 25, 50 μM) could dose-dependently inhibit PDGF-BB-induced VSMC proliferation. Its anti-proliferative effects may be mediated by preventing cell cycle progression, regulating protein expression of cell cycle regulators, and inhibiting ERK1/2 phosphorylation (Xu et al., 2011). Similarly, TSG (10, 50, 100 μM) also had a concentration-dependent inhibitory effect on angiotensin II (Ang II)-induced VSMC proliferation (Xu et al., 2015). According to the results of biochemical experimental studies such as flow cytometry and Western blotting, the effect might be related to the down-regulation of intracellular ROS and the inhibition of the Src-MEK1/2-ERK1/2 signaling pathway, which blocked cell cycle progression (Xu et al., 2015). A subsequent study showed that TSG (30, 60, 120 mg/kg) reduced the cardiac remodeling in pressure-overloaded rats in an abdominal aortic banding-induced cardiac remodeling model (Xu et al., 2014). With subsequent in-depth studies, the protective mechanisms may be related to reducing Ang II levels, enhancing SOD and GSH-Px activities in serum and cardiac tissue, and inhibiting TGF-β1 protein expression as well as ERK1/2 and p38 phosphorylation (Xu et al., 2014).

#### 3.4.4 VEGF/ICAM-1

Studies have reported that high concentrations of vascular endothlial growth factor (VEGF) -A are detected in some cardiovascular diseases and are often associated with poor prognosis and disease severity (ErŽen et al., 2014). Therefore, controlling angiogenesis and VEGF-A may improve the life quality and longevity of patients with cardiovascular diseases. A study showed that TSG could promote reendothelialization by increasing the levels of serum VEGF and the expression of CD34 in the vessel wall (Hu et al., 2019). In addition, intercellular cell adhesion molecule-1 (ICAM-1), also known as CD54, is an important adhesion molecule mediating adhesive responses (Bui et al., 2020). Enhanced expression of ICAM-1 is considered as an important marker of atherosclerotic lesions (Watanabe and Fan, 1998). Thus, ICAM-1 can be exploited to identify the development and prognosis of atherosclerosis. Yang et al. (2005) showed that stilbene glycoside from PM had anti-atherosclerotic effects by inhibiting ICAM-1 and VEGF expression in foam cells. Subsequently, the study by Wang et al. (2013) further confirmed that TSG could inhibit the expression of adhesion molecules (ICAM-1/VCAM-1) in both *in vitro* (oxLDL-induced U937 cells) and *in vivo* (the aortic wall of rats with diet-induced atherosclerosis) models. However, the mechanism by which TSG orchestrates this effect is unknown, remaining more thorough experimental research.

#### 3.4.5 RhoA/ROCK

Accumulating evidence from basic and clinical studies suggests a pivotal role of RhoA/ROCK signaling in the pathogenesis of a variety of cardiovascular diseases, including essential hypertension, congestive heart failure, coronary heart disease, and atherosclerosis (Cai et al., 2016). Among them, increased ROCK activity mediates VSMC hypercontractility, endothelial dysfunction, inflammatory cell recruitment, and vascular remodeling (Surma et al., 2011). A recent study showed that TSG (25, 50, 100 μM) could ameliorate LPS-induced endothelial dysfunction by inhibiting RhoA/ROCK signaling (Qi et al., 2020). It is well known that the reorganization of F-actin in endothelial cells is an important pathological factor leading to increased endothelial permeability and endothelial dysfunction (Shi et al., 2013). Interestingly, the team also found that TSG regulated F-actin cytoskeletal rearrangement by inhibiting the RhoA/ROCK signaling pathway, thus inhibiting the formation of contractile ring and the changes of cell morphology (Qi et al., 2020).

#### 3.4.6 Autophagy

Under normal conditions, the level of autophagic activity plays an important role in maintaining the homeostasis and function of cells. In particular, autophagy contributes to the maintenance of intracellular homeostasis in most cardiovascular cells, including cardiomyocytes, endothelial cells, and arterial smooth muscle cells (Bravo-San Pedro et al., 2017). However, defective or excessive autophagy may contribute to cardiovascular diseases such as atherosclerosis, heart failure, and hypertension (Mialet-Perez and Vindis, 2017). Dong et al. (2016) reported that TSG could inhibit excessive autophagy and improve microvascular endothelial dysfunction in a rat model of spontaneous hypertension. The protective effect was mainly attributed to the restoration of microvascular endothelial dysfunction by activating the AKT/mTOR pathway, which inhibited autophagy (Dong et al., 2016). This suggests that TSG can be applied to protect vascular function against subclinical changes in prehypertension.

#### 3.4.7 MicroRNAs

It is reported that microRNA plays a key regulatory role in some important cellular pathways and the occurrence and development of various diseases (Liu et al., 2014). In cardiovascular diseases, it can play a cardiovascular protective role by regulating cardiomyocytes, fibroblasts and endothelial cells (Kalayinia et al., 2021). For example, a study showed that miR-129-3p may be involved in angiotensin II-mediated cardiac biology and diseases (Jeppesen et al., 2011). In addition, a genome-wide expression study of circulating microRNAs in patients with heart failure showed that the level of miR-129-3p in serum was significantly reduced (Cakmak et al., 2015). These results suggested that miR-129-3p might be a susceptible gene associated with cardiovascular disease, which was inhibited under the pathological conditions of myocardial cells. In one study, TSG (0.4 mM) treatment could reduce the palmitic acid-induced myocardial cells inflammation and apoptosis (Zou and Kong, 2019). According to the results of biochemical pharmacology and bioinformatics, its protective effect on cardiomyocytes may be mediated by targeting the miR-129-3p/Smad3 signaling pathway, including up-regulating the expression of miR-129-3p and inhibiting the expression of p-Smad3 (Zou and Kong, 2019). These results show that microRNA, as an important regulatory factor in the body, can regulate different molecular networks by interfering with the target genes, thus showing the effect of cardiovascular protection.

#### 3.4.8 Others

Doxorubicin, also known as adriamycin, is an anthracycline antibiotic commonly used to treat a variety of cancers (Rivankar, 2014). However, severe side effects, such as cardiomyopathy and heart failure, have been observed in patients receiving doxorubicin treatment, which greatly limit its clinical application (Octavia et al., 2012). Interestingly, a study showed that TSG could reduce doxorubicin-induced cardiotoxicity *in vitro* (cardiomyocytes from neonatal Wistar rats) and *in vivo* (Kunming male mice) (Zhang et al., 2009a). With more in-depth studies, it has been shown that its cardioprotective effects are mainly achieved by inhibiting ROS production, the increase of intracellular Ca^2+^, and apoptosis-related signaling pathways (Zhang et al., 2009a). From these aspects, TSG may be a promising agent combined with doxorubicin to attenuate cardiotoxicity and improve its clinical efficacy. In addition, Peng et al. (2016) showed that TSG (120 mg/kg) also had beneficial effects on pressure overload-induced cardiac fibrosis. The protective effect on the heart was mainly attributed to the up-regulation of endogenous PPAR-γ, a potent endogenous antifibrotic factor (Peng et al., 2016).

Furthermore, Yao et al. (2013) investigated the protective effects of TSG on atherosclerotic rats by proteomic analysis. The results showed that a total of 21 proteins were involved in the anti-atherosclerotic effects of TSG, including HSP70, lipocortin 1, Apo A-I, calreticulin, vimentin, and so on, which were closely related to inflammation, cholesterol transport, apoptosis, and adhesion (Yao et al., 2013). However, the specific molecular mechanisms by which TSG regulated the expression of these proteins are not well defined. Subsequently, the study of Chen et al. (2018b) confirmed that TSG could also attenuate atherosclerosis in apolipoprotein E-deficient mice by promoting reverse cholesterol transport. Specifically, TSG achieved its protective effect on arteriolosclerosis by promoting the cholesterol efflux of macrophages and SR-BI-mediated hepatic cholesterol uptake, increasing the secretion of cholesterol from ABCG5 into bile, and improving cholesterol metabolism *via* CYP7A1 (Chen et al., 2018b).

### 3.5 Hepatoprotective Effects

TSG has been reported for the treatment of various liver diseases, such as alcoholic fatty liver disease, non-alcoholic fatty liver disease (NAFLD) and non-alcohol steatohepatitis (NASH). For example, TSG (25, 50, 100 μg/ml) significantly improved high cholesterol diet-induced NAFLD in zebrafish larvae (Wang et al., 2020a). According to the results of biochemical pharmacology, the mechanisms of its anti-NAFLD effect are mainly attributed to the regulation of the lipid metabolism-related pathways, the anti-inflammatory pathway IKKβ/NF-κB and the antioxidant pathway Keap1-Nrf2 (Wang et al., 2020a). Similarly, TSG (50, 100 mg/kg) was first demonstrated to significantly improve NASH and atherosclerosis in the mouse models with high fat diet (HFD)-induced metabolic syndrome and NASH (Xu et al., 2019). Its hepatoprotective effect was at least partly dependent on modulating the key regulators of lipid metabolism, inflammation, fibrosis and oxidative stress (Xu et al., 2019). In addition, Zhang et al. (2020) found TSG had a good ameliorative effect on alcoholic hepatic steatosis by *in vitro* (THP-1 macrophages) and *in vivo* (C57BL/6 mice) experiments. Specifically, TSG inhibits P2X7R-NLRP3 signaling in macrophages, and is subsequently unperturbed by activated macrophages, thus inhibiting the lipid accumulation in hepatocytes to attenuate alcoholic hepatic steatosis (Zhang et al., 2020). In conclusion, the regulation of alcoholic hepatic steatosis by TSG may be achieved by targeting the crosstalk between macrophages and hepatocytes.

In recent years, increasing evidence has demonstrated that gut microbiota plays a crucial role in the pathogenesis of liver diseases (Albhaisi et al., 2020). Briefly, when gut microbiota is disrupted or dysregulated, the bacteria in the gut and their metabolites such as endotoxin and LPS, can be transferred through the damaged intestinal tight junctions in the portal circulation, thereby activating cytokines *via* toll-like receptors and releasing IL-8 to stimulate the inflammatory response, which may contribute to liver inflammation and fibrosis (Henao-Mejia et al., 2012; Stevens et al., 2018). Interestingly, the study by Lin et al. (2015b) showed that, in HFD-induced NAFLD rats, TSG (12, 24, 48 mg/kg) could improve intestinal mucosal barrier function by regulating the balance of intestinal flora as well as increasing the protein expression of ZO-1 and Occludin, and then reverse the occurrence and development of NAFLD. These results suggest that TSG may serve as a promising drug candidate for the prevention and intervention of NAFLD/NASH.

### 3.6 Anti-Osteoporosis Effects

Despite the availability of drugs to treat osteoporosis, osteoporosis currently remains a huge and growing public health problem due to the certain rare side effects of these drugs and the lack of clear evidence supporting their long-term efficacy (Khosla and Hofbauer, 2017). Therefore, new drugs with no side effect and long-term anabolic effects on bone are need urgently. In recent years, there have been research showing that TSG has the ability to promote bone formation as well as protect osteoblasts, with a greater potential in the treatment of osteoporosis. Zhang et al. (2012) showed that TSG (0.1, 1, 10 μM) pretreatment for 24 h protected against H_2_O_2_-induced dysfunction and oxidative stress in osteoblastic MC3T3-E1 cells. Its protective effect is mainly mediated by inhibiting the release of bone resorption mediators and oxidative damage to cells (Zhang et al., 2012). Similarly, in MC3T3-E1 cells and bone tissue of BALB/c mouse model, TSG may also achieve preventive and protective effects against osteoporosis *via* PI3K/AKT, Wnt/FoxO3a, immune system and chemokine signaling pathway (Zhang et al., 2018a; Fan et al., 2018b).

Emerging evidence suggests that an increase in the activity of the skeletal renin-angiotensin system (RAS) is detrimental to bone tissue during hyperglycemia (Yamamoto et al., 2015; Zhang et al., 2016). In addition, it has been shown that TSG blocks the activity of tissue RAS, which plays a key role in the development of diabetic osteoporosis (Skov et al., 2014). Interestingly, the study of Zhang et al. (2019) confirmed that TSG (10, 40 mg/kg) had a protective effect on streptozotocin-induced diabetic osteoporosis in hyperglycemic mice, which might be attributed to its regulation of bone formation and bone resorption by inhibiting local RAS and acting on osteoblasts and osteoclasts (Zhang et al., 2019). However, whether TSG can ameliorate other tissue damage caused by local activation of RAS or directly act on RAS require further investigation. Furthermore, in bone marrow mesenchymal stem cells of rats and the dexamethasone-induced zebrafish model of osteoporosis, different doses of TSG were shown to promote osteoblast differentiation, as indicated by enhanced alkaline phosphatase activity, increased osteocalcin content, and increased nodule area (Zheng et al., 2017). Taken together, TSG may serve as a good drug candidate for the treatment of osteoporosis.

### 3.7 Memory-Enhancing Effects

It has been reported that TSG (1, 5, 10 μM) can improve and modify neuronal networks by increasing the activity of CaMKII and ERK1/2 in the hippocampus of normal mice and promoting hippocampal long-term potentiation (LTP), which enhances their learning and memory abilities (Wang et al., 2011). Similarly, the study by Zhou et al. (2012) suggested that the protective effect of TSG (50 mg/kg) on learning and memory impairment might be achieved by improving the structure and function of neuronal synapses through the up-regulation of SRC and p-NR2B. In addition, the study of Chen et al. (2016) showed that TSG (20, 40, 80 mg/kg) could promote hippocampal memory and synaptic plasticity in normal mice. Specifically, TSG enhances hippocampus-dependent contextual fear memory and novel object recognition, promoting hippocampal LTP and increasing dendritic spine density in the CA1 region (Chen et al., 2016). According to their further research, the mechanism that TSG enhanced memory might be related to the phosphorylation of ERK1/2, CaMKII, cAMP-response element binding protein (CREB) in the hippocampus, the up-regulation of BDNF expression, and the activation of the SIRT1/miR-134 signaling pathway (Chen et al., 2016).

Numerous studies on animals and humans have shown that cognitive decline is related to the accumulation of neurotoxin Aβ in the aging brain, which is produced by the proteolytic cleavage of APP (Kikuchi et al., 2011). Therefore, APP cleaving enzymes such as a disintegrin and metalloprotease (ADAM) 10 and 17, may be effective targets for reducing Aβ peptide formation and function and ameliorating the cognitive deficits associated with aging (Chow et al., 2010). Interestingly, the study by Hou et al. (2011) confirmed that TSG (50 mg/kg) could improve learning and memory ability of rats via the APP pathway. The further study showed that the effect is at least partly mediated by selectively promoting ADAM10 expression (Hou et al., 2011). Moreover, in APP695V717I-transgenic mice, TSG (100 mg/kg) could also alleviate Aβ neurotoxin-induced functional impairment of the endoplasmic reticulum by reducing the expression of Beclin-1 and LC3-Ⅱ in autophagy pathway, which improves the learning, memory, and spatially oriented behavior in mice (Luo et al., 2015).

Furthermore, Zhang et al. (2006) showed that TSG (120, 240 μM/kg) not only prevented the learning and memory deficits at the early stage of AD-like model, but also reversed them at the late stage. Seven years later, the team found that the protective effect of TSG might be closely related to inhibiting the overexpression of α-synuclein in APP-transgenic mice (Zhang et al., 2013c). Because TSG can not only inhibit α-synuclein overexpression at an early stage, but also reverse the increased expression, which inhibits its subsequent aggregation (Zhang et al., 2013c). Taken together, these results suggest that TSG supplementation may have clinical implications for the treatment of the diseases associated with cognitive deficits.

### 3.8 Anti-Aging Effects

Cellular senescence refers to the growth arrest due to various cellular stresses, including DNA damage, inadequate mitochondrial function, activated oncogenes, and oxidative stress (Bellei and Picardo, 2020). Meanwhile, cellular senescence is also an important mechanism to protect cells from oncogenic stress (Campisi and d'Adda di Fagagna, 2007). Among them, skin aging is a typical manifestation in aging mammals or humans. Its extent depends on a high content of collagen fibers, natural elastic fibers, and a slowly thinning dermal layer, which all lead to the skin aging symptoms such as wrinkling and skin laxity (Quan et al., 2012; Velarde et al., 2012). A report showed that TSG (180 mg/kg) treatment increased collagen fibers, thickened dermal layers, and decreased insulin and insulin-like growth factor-1 (IGF-1) levels in the skin of aging mice (Zhou et al., 2014). Further studies have shown that the mechanism of its anti-cutaneous aging effect is mainly through inhibiting the insulin/IGF-1 signaling pathway (Zhou et al., 2014). In the angiotensin II-induced senescence model of HUVECs, TSG (20, 40 μg/ml) treatment decreased the percent of senescence-associated-β-galactosidase positive cells and the expression levels of cellular senescence biomarkers, p53 and PAI-1 protein (Fan et al., 2021). Its anti-aging mechanism is mainly attributed to the regulation of SIRT1 activity (Fan et al., 2021). Similarly, in the mouse model established by high calorie diet, TSG delayed the aging of aged mice consuming excess calories (Ning et al., 2018). The mechanism may be related to regulating the AMPK/SIRT1/PGC-1α signaling pathway to improve motor function, bone mineral density, and organ protection in the aging mice (Ning et al., 2018).

It has been reported that enhancing the expression of Klotho, a type I transmembrane protein closely related to aging, can retard the aging process (Kuro-o, 2008). Interestingly, Zhou et al. (2015) demonstrated that TSG (2, 20, 50 μM) had an anti-aging effect on senescence-accelerated mouse prone 8 (SAMP8). According to the findings of biochemical pharmacology, the protective mechanism may be associated with the up-regulation of neural Klotho and down-regulation of neural insulin or IGF-1 levels (Zhou et al., 2015). In addition, the study by Shen et al. (2015) showed that TSG (50, 100, 200 mg/kg) could also significantly improve memory and motor function in aged mice. Specifically, TSG treatment significantly protected synaptic ultrastructure, increased the number of synaptic connections and the levels of synapse-related proteins (synaptophysin, phosphorylated synaptophysin I, and postsynaptic density protein 95), and suppressed the overexpression of α-synuclein in the hippocampus, striatum, and cerebral cortex of aged mice (Shen et al., 2015). Therefore, TSG may serve as a potential candidate for preventing aging and treating aging-related neurodegenerative diseases.

### 3.9 Other Pharmacological Effects

TSG (10, 20 mg/kg) significantly reduced TC, TG, blood urea nitrogen, creatinine, 24 h urinary protein, and MDA levels, as well as significantly increased SOD and GSH-Px activities in rats with diabetic nephropathy (Li et al., 2010a). Moreover, TSG treatment also significantly suppressed the overexpression of TGF-β1 and COX-2 in diabetic rats, and restored the decrease of SIRT1 expression (Li et al., 2010a). Its protective mechanisms against diabetic nephropathy may involve attenuating oxidative stress damage, inhibiting inflammation, and regulating SIRT1 and TGF-β1 signaling pathway (Li et al., 2010a). A recent study showed that TSG (12 mg/kg) also ameliorated traumatic brain injury by attenuating oxidative stress, inflammation and apoptosis in the mouse model (Cao et al., 2020). The protective effect may be achieved by inhibiting the Ras/JNK signaling pathway (Cao et al., 2020).

It was reported that TSG (10, 20, 40, 80 mg/kg) could prolong sleep duration and prevent insomnia in mice (Wei et al., 2017). The effect may be closely related to the regulation of sleep time by affecting the activities of lactate dehydrogenase and salivary amylase (Wei et al., 2017). Furthermore, Wang et al. (2017) used forced swim test, suspended tail test, and chronic social defeat stress depression model to explore the antidepressant effect of TSG. The results showed that TSG (30, 60 mg/kg) exerted antidepressant-like effects in mice by enhancing the hippocampal BDNF system (Wang et al., 2017). Interestingly, it has also been shown that TSG (200 μM) has a significant hair-regeneration effect (Chen et al., 2018a). Because TSG could inhibit the p53-, Fas-, and Bax-induced apoptotic signaling pathways, thus preventing hair follicles from entering the catagen phase (Chen et al., 2018a).

In addition, the study of Lin et al. (2018) showed that TSG enhanced the self-renewal of human dental pulp stem cells (HDPSCs). Specifically, TSG (10 μM) treatment not only enhanced cell viability, colony formation rate, and telomerase activity of HDPSCs, but also significantly increased the mRNA expression of proliferation markers, including the NAD^+^-dependent histone deacetylase SIRT1, ribonucleotide reductase subunit M2, proliferating cell nuclear antigen, and cyclin D1 (Lin et al., 2018). In addition, TSG treatment could also increase the expression of proliferation-related proteins and pluripotent stem cell markers, including AMPK, ERK, and so on. (Lin et al., 2018). The beneficial effect may be achieved by enhancing the renewal capacity and proliferative potential of HDPSCs through the AMPK/ERK/SIRT1 axis (Lin et al., 2018). The effects of TSG on enhancing the osteodifferentiation potential and molecular regulation of HDPSCs deserve more in-depth investigation, as it may provide a novel autologous cell-based therapeutic strategy for regenerative dentistry.

## 4 Toxicity of TSG

At present, most experiments have proved that TSG is not obviously toxic to cells or experimental animals. For example, the study by Yu et al. (2011) showed that TSG (20–300 μM) had no cytotoxicity for the human hepatocyte cell line LO_2_ in terms of cell proliferation, cell membrane integrity and enzyme secretion. Although TSG with a single use was not significantly toxic, a few studies have shown that TSG enhances the toxic effects of other drugs. For example, emodin, which has been shown to have significant hepatotoxicity, is metabolically eliminated *in vivo* mainly through a phase II metabolic reaction. However, the study by Ma et al. (2013) showed that TSG (117 mg/kg) inhibited emodin elimination *in vivo* by down-regulating UDP glucuronosyltransferase 1A8, which leaded to emodin accumulation, thus exhibiting liver toxicity. Similarly, the study by Xu et al. (2017) showed that TSG (100–800 mg/kg) could exacerbate acetaminophen-induced hepatotoxicity because it increased the expression and catalytic activity of hepatic CYP2E1, CYP3A4, and CYP1A2. In contrast, the serum ALT/AST activity assay and liver histopathology result showed that the single use of TSG (100–800 mg/kg) did not induce significant pathological changes (Xu et al., 2017). This suggested that there might be interactions between different ingredients, which affected the related drug metabolizing enzymes. Therefore, it is important to pay special attention to the combined use of TSG with other drugs in clinic. Moreover, a comprehensive and systematic toxicology study is needed in order to access the toxicity and mechanism of action of TSG.

## 5 Pharmacokinetic Study of TSG

Nowadays, pharmacokinetic research is in an equally important position as pharmacodynamic and toxicological research, having become an important part of preclinical and clinical research of drugs. At the same time, it plays an important role in the creation of new drugs, the improvement of dosage forms, and the study of the mechanism of dosage forms (Li et al., 2015). The method of current pharmacokinetic studies on TSG is mainly using high performance liquid chromatography tandem mass spectrometry (HPLC-MS/MS) and ultra performance liquid chromatography tandem mass spectrometry (UPLC-MS/MS) to analyze plasma samples from rats (Zhang et al., 2018b; Shen et al., 2018; Ma et al., 2021). A comparison of the pharmacokinetic parameters of TSG from 5 studies is shown in Table 3.

**TABLE 3:** Pharmacokinetic studies of TSG.

Route of administration	Species	Dose (mg/kg, equivalent to TSG)	Pharmacokinetic parameters								References
			T_max_ (a: h; b: min)	AUC_0-t_ (a: ng.h/ml; b: mg.h/L; c: μg.min/ml)	AUC_0-∞_ (a: ng.h/ml; b: mg.h/L; c: μg.min/ml; d: μg.h/L)	t_1/2_ (h)	V_d_ (L/kg)	C_L_ (a: L/min.kg; b: L/h.kg)	C_max_ (a: ng/ml; b: mg/L; c: μg/ml; d: μg/L)	MRT_0-t_ (a: h; b: min)	
Oral	Rats (male, SD)	212.23	0.25 ± 0.14 (a)	80.39 ± 34.36 (a)	90.24 ± 35.81 (a)	2.21 ± 1.60	N/A	N/A	69.62 ± 51.79 (a)	3.92 ± 3.48 (a)	Zhang et al. (2018b)
		156.35	0.30 ± 0.07 (a)	134.08 ± 94.04 (a)	137.77 ± 93.26 (a)	0.90 ± 0.68	N/A	N/A	211.39 ± 100.81 (a)	1.03 ± 0.41 (a)	
		34.23	0.28 ± 0.14 (a)	1.2 ± 0.92 (a)	1.14 ± 1.05 (a)	0.12 ± 0.05	N/A	N/A	7.83 ± 8.04 (a)	0.29 ± 0.05 (a)	
Oral	Rats (male, SD)	75	0.5000 ± 0.0021 (a)	0.5099 ± 0.2907 (b)	0.6399 ± 0.3472 (b)	N/A	N/A	N/A	0.3495 ± 0.1513 (b)	1.3348 ± 0.3875 (a)	Sun et al. (2018)
i.v.	Rats (male, SD)	10	N/A	307.0 ± 20.0 (c)	350.5 ± 11.5 (c)	N/A	3.4 ± 1.7	0.03 ± 0.001 (a)	22.8 ± 2.6 (c)	N/A	Zhao et al. (2013)
		20	N/A	660.4 ± 34.7 (c)	734.0 ± 59.0 (c)	N/A	7.0 ± 2.1	0.03 ± 0.002 (a)	64.2 ± 3.6 (c)	N/A	
Oral	Rats (male, SD)	50	14.8 ± 1.2 (b)	359.2 ± 35.6 (c)	425.5 ± 112.2 (c)	N/A	14.1 ± 4.4	0.12 ± 0.03 (a)	5.7 ± 1.6 (c)	N/A	Zhao et al. (2013)
		100	14.2 ± 1.1 (b)	1,242.5 ± 38.4 (c)	1,277.5 ± 37.7 (c)	N/A	3.9 ± 0.5	0.08 ± 0.002 (a)	21.9 ± 2.5 (c)	N/A	
Oral	Rats (male, SD)	1.92	0.50 ± 0.31 (a)	283.8 ± 189.1 (a)	297.5 ± 185.3 (a)	1.99 ± 1.31	N/A	N/A	99.1 ± 27.1 (a)	N/A	Shen et al. (2018)
Oral	Rats (male, SD)	3,640	0.25 ± 0.0293 (a)	N/A	12,860.002 ± 1,267.111 (d)	1.947 ± 0.806	4.369 ± 0.711	1.555 ± 0.150 (b)	15,042.977 ± 1,420.870 (d)	4.540 ± 1.225 (a)	Ma et al. (2021)

Oral: oral administration; i.v.: intravenous administration; N/A: not applicable.

The plasma concentration-time profile of TSG after its oral and intravenous administration was fitted to a two-compartment pharmacokinetic model in rats (Zhao et al., 2013). The study by Zhao et al. (2013) showed that TSG was rapidly absorbed and eliminated in rats, as it was detected in plasma at 2 min. Similarly, it was also confirmed in the study of Shen et al. (2018). After oral administration of 100 mg/kg TSG to rats for 30 min, the heart (1,324 μg/g) and kidney (1,155 μg/g) are the main tissues with preferential distribution of TSG, followed by liver, lung, and stomach (295–654 μg/g), with the least distribution in brain and intestine (Zhao et al., 2013; Li et al., 2020a). Differently, after intravenous administration of 10 mg/kg TSG for 10 min, the liver is the main organ with preferential distribution of TSG (89.75 μg/g), followed by heart, lung, spleen, kidney, stomach, small intestine, and brain (7.56–37.15 μg/g), with the least distribution in testis (Zhao et al., 2013; Li et al., 2020a). The C_max_ values of TSG after oral administration (50, 100 mg/kg) were 5.7 ± 1.6 and 21.9 ± 2.5 μg/ml respectively, whereas they were 22.8 ± 2.6 and 64.2 ± 3.6 μg/ml respectively after intravenous administration (10, 20 mg/kg), which indicated that the plasma level of TSG was much higher after intravenous administration than oral administration (Zhao et al., 2013). Recent preclinical studies have shown that phase II metabolism is the main metabolic pathway of TSG (Li et al., 2020a). After incubating TSG with liver microsomes of rats for 1 h, only TSG glucuronide was determined (Wang et al., 2009a). After oral administration of TSG in rats, its glucuronidation metabolites were also identified as the main metabolites (Zhao et al., 2013). In addition, a study showed that the absolute oral bioavailabilities of TSG with a low dose (50 mg/kg) and a high dose (100 mg/kg) were 24.2 and 36.5% respectively (Zhao et al., 2013). Taken together, these studies about pharmacokinetics and bioavailability suggest that TSG is rapidly absorbed and eliminated, and may have a wide biological distribution after oral administration in normal rats.

Due to the poor stability *in vitro*, poor absorption in the intestine, and only short-term storage in tissues, some researchers focus on improving the stability and bioavailability of TSG through numerous experiments. For example, Sun et al. (2018) showed that *Ophiopogon japonicus* (Thunb.) Ker Gawl. polysaccharides (OJPs) could significantly improve the water solubility and stability of TSG. Significantly, the T_max_, C_max_, and AUC_(0-tn)_ values of TSG-OJP were respectively 3.5-, 1.45-, and 2.32-fold higher than those of TSG, indicating that OJPs can be used to improve the biopharmaceutic properties of TSG as well as enhance its pharmacological effects (Sun et al., 2018). In conclusion, it is expected that follow-up pharmacokinetic studies of TSG can be performed in multiple animals, which may provide useful information for the development of potential new dosage forms and the clinical use of drugs.

## 6 Discussion and Future Perspective

PM, one of the most widely used traditional Chinese medicines for thousands of years, is often used clinically to treat hyperlipidemia, coronary heart disease, stroke, alopecia as well as aging disorders such as aged white hair, tooth loss, and senile plaques. As the main and unique active index ingredient of PM, TSG has various pharmacological effects, including anti-inflammation, antioxidation, neuroprotection, cardiovascular protection, hepatoprotection, anti-osteoporosis, memory enhancement, antidepressant, and anti-aging. Therefore, TSG plays an important role in the treatment of many diseases involving inflammatory diseases, neurodegenerative diseases, cardiovascular diseases, liver injury, osteoporosis, diabetic nephropathy, depression, and aging.

For inflammatory diseases, TSG regulates the NF-κB, AMPK/Nrf2, NLRP3 inflammasome, p38 MAPK, and ERK1/2 signaling pathways to exhibit significant anti-inflammatory effects on various inflammatory models. In addition, TSG has significant scavenging ability for free radicals and can exert excellent antioxidant effects by regulating oxidative damage markers (SOD, CAT, GSH-Px), inhibiting the overproduction of ROS, NO, and lipid peroxidation products, decreasing apoptosis, alleviating autophagy, and enhancing the endogenous antioxidant defense system by activating the Nrf2 antioxidant pathway. Interestingly, due to its significant anti-inflammatory and antioxidant activities, TSG exerts neuroprotective and cardiovascular protective effects through multiple molecular mechanisms. In terms of the neuroprotective effects, through modulating the Bcl-2/Bax/Caspase-3, NF-κB, MAPK, PI3K-AKT, JNK, Wnt/β-Catenin, keap1-Nrf2, and AMPK/PINK1/Parkin signaling pathways, TSG shows excellent neuroprotective effects against neurodegenerative diseases such as AD, PD, and cerebral ischemic injury. Regarding its cardiovascular protective effects, TSG shows significant protective effects against various cardiovascular diseases by regulating the TGF-β/Smad, eNOS/NO, Src-MEK1/2-ERK1/2, NO/cGMP/PKG, VEGF/ICAM-1, and RhoA/ROCK signaling pathways as well as autophagy and microRNAs.

The anti-inflammatory and antioxidant pharmacological activities of TSG also contribute to its hepatoprotective effects, making TSG a promising candidate for the treatment of liver diseases, such as alcoholic fatty liver disease, NAFLD, and NASH. This is attributed primarily to the regulation on lipid metabolism, IKKβ/NF-κB, keap1-Nrf2, and P2X7R-NLRP3 signaling pathways as well as gut microbiota and intestinal mucosal barrier function. Furthermore, TSG achieves preventive and protective effects against osteoporosis by modulating the PI3K/AKT, Wnt/FoxO3a, immune system, and chemokine signaling pathways as well as blocking the tissue RAS activity. Interestingly, TSG can also enhance learning and memory abilities by promoting hippocampal long-term potentiation, improving the structure and function of neuronal synapses, decreasing the expression of Beclin-1 and LC3-II in the autophagy pathway, and alleviating Aβ neurotoxin-induced functional impairment of the endoplasmic reticulum. More importantly, TSG can serve as a potential candidate for aging prevention and the treatment of aging-associated diseases, which is mainly attributed to regulating the insulin/IGF-1 and AMPK/SIRT1/PGC-1α signaling pathways. In addition, TSG shows protective effects against diabetic nephropathy, traumatic brain injury, depression, and alopecia. In summary, TSG has broad and significant pharmacological activities, and is a potential compound for the treatment of many diseases.

Most experiments have confirmed that TSG is not obviously toxic to cells and experimental animals, but its combined use with other toxic drugs may affect the related drug metabolizing enzymes, thus leading to highly toxic side effects that should be noted. It is worth mentioning that TSG exists as *cis*- and *trans*-isomers. Although TSG mostly exists in *trans*-structure in nature, TSG is optically sensitive, and the conversion of *trans*-structure to *cis*-structure occurs during traditional processing steps or under UV irradiation, leading to an increased content of cis TSG. *Trans*-TSG and *cis*-TSG have been reported to differ in their pharmacological effects, toxicity, and pharmacokinetic properties. However, most of the published articles on TSG do not clearly indicate its specific structure in research, which makes it difficult to distinguish and compare the activities of the two isomers. Therefore, it is necessary to remind other scholars studying TSG to clearly indicate the specific structure of TSG when publishing their articles, which may provide direction and basis for the further development and clinical application of TSG.

The pharmacokinetic results of TSG vary because of the different route and dosage of administration, and different experimental models. In general, pharmacokinetic studies showed that the absorption and elimination of TSG *in vivo* is very rapid and has a wide biological distribution. Although TSG has been proved to be a promising compound with multiple pharmacological effects, it also has some disadvantages, such as poor stability *in vitro*, poor intestinal absorption, and short-term storage in tissues. Therefore, we propose the following views. First of all, in subsequent studies, we can combine omics research methods, such as transcriptomics/proteomics/metabolomics, to screen out the differentially expressed genes/proteins/metabolites, and further clarify the molecular mechanism by which TSG exerts multiple pharmacological effects as well as the specific targets. Secondly, it is suggested to add more randomized controlled clinical trials on TSG in the future to fully evaluate its actual clinical efficacy and its safety in clinical application. In addition, in view of the poor stability and low bioavailability of TSG, in the future, we should focus on modifying its chemical structure and developing its new dosage forms, so as to improve its stability and bioavailability, which provides a wider range for the clinic use of TSG. It is worth mentioning that, in recent years, there are many reports that liposomes and exosomes can effectively improve the targeting effect and bioavailability of drugs (Liao et al., 2019; Guimarães et al., 2021). Therefore, in follow-up studies, we can further explore how to increase the stability and bioavailability of TSG through the targeted delivery of liposomes and exosomes *in vivo*. In summary, this study provides a comprehensive review and summary of the physical/chemical properties, pharmacological activity, toxicity, and pharmacokinetic properties of TSG, with a view to providing a theoretical basis for the development and clinical application of TSG.

## Glossary

PM: *Polygonum multiflorum* Thunb.
TSG: 2,3,5,4′-tetrahydroxystilbene-2-O-β-D-glucoside
NF-κB: nuclear factor kappa-B
AMPK: adenosine 5′-monophosphate (AMP)-activated protein kinase
PI3K: phosphatidyl-inositol-3-kinase
AKT: protein kinase B
ROS: reactive oxygen species
NO: nitric oxide
Bcl-2: B-cell lymphoma-2
Bax: Bcl-2-associated X protein
TGF-β: transforming growth factor-β
PGE2: prostaglandin E2
iNOS: inducible nitric oxide synthase
eNOS: endothelial nitric oxide synthase
COX-2: cyclooxygenase-2: cyclooxygenase-2
LPS: lipopolysaccharide
IL-6: interleukin-6
TNF-α: tumor necrosis factor-α
IL-1β: interleukin-1β
SIRT1: sirtuin 1
MPO: myeloperoxidase
MDA: malonaldehyde
SOD: superoxide dismutase
COX-2: cyclooxygenase-2: cyclooxygenase-2
PPAR-γ: peroxisome proliferator-activated receptor γ
CAT: catalase
GSH-Px: glutathione peroxidase
Nrf2: nuclear factor erythroid 2-related factor 2
HO-1: heme oxygenase 1
GST: glutathione-s-transferase
AD: Alzheimer’s disease
PD: Parkinson’s disease
Aβ: amyloid β-protein
MPP+: 1-methyl-4-phenylpyridinium
MAPK: mitogen-activated protein kinase
CNS: central nervous system
MCAO: middle cerebral artery occlusion
GLT-1: glutamate transporter-1
JNK: Jun N-terminal kinase
PINK1: PTEN induced putative kinase 1
ROCK: Rho kinase
NGF: nerve growth factor
GAP-43: growth associated protein-43
APP: β-amyloid precursor protein
BACE1: β-site APP cleaving enzyme 1
PS1: presenilin 1
BDNF: brain-derived neurotrophic factor
TrkB: tropomyosin-related kinase B
HUVECs: human umbilical vein endothelial cells
oxLDL: oxidized low-density lipoprotein
PMA: phorbol-12-myristate-13-acetate
VSMC: vascular smooth muscle cells
ERK1/2: extracellular signal-regulated kinase 1/2
AngII: angiotensin II
VEGF: vascular endothlial growth factor
ICAM-1: intercellular cell adhesion molecule-1
NAFLD: non-alcoholic fatty liver disease
NASH: non-alcohol steatohepatitis
HFD: high fat diet
RAS: renin-angiotensin system
LTP: long-term potentiation
CREB: cAMP-response element binding protein
ADAM: a disintegrin and metalloprotease
IGF-1: insulin-like growth factor-1
SAMP8: senescence-accelerated mouse prone
HDPSCs: human dental pulp stem cells
HPLC-MS/MS: high performance liquid chromatography tandem mass spectrometry
UPLC-MS/MS: ultra performance liquid chromatography tandem mass spectrometry
